# Supplementary material for: Emergent cooperative superstructures via order-disorder kinetics in molecular intercalation superlattices
Source: Sci Adv. 2026 Aug 5;12(32):eaeg4785. doi: 10.1126/sciadv.aeg4785 (PMC13440418; doi:10.1126/sciadv.aeg4785)
Supplement: Supplementary file 1 [file sciadv.aeg4785_sm.pdf]

Science Advances
AAAS

Supplementary Materials for

Emergent cooperative superstructures via order-disorder kinetics in molecular intercalation superlattices

Taiga Ueda *et al.*

Corresponding author: Hideki Matsuoka, hideki-m@iis.u-tokyo.ac.jp

Sci. Adv. **12**, eaeg4785 (2026)
DOI: 10.1126/sciadv.aeg4785

This PDF file includes:

Supplementary Text
Figs. S1 to S12
Tables S1 to S5
References

Supplementary Text

Sample information and details of the intercalation method

This section provides detailed information on the samples used in this study and the procedures employed for electrochemical intercalation. A summary of all samples is presented in Tables S1 and S2. The intercalation was performed using three different setups (A–C), described below and illustrated in Fig. S6.

Setup A: This configuration follows the previously reported electrochemical intercalation setup for $NbSe_2$(*17*). As shown in Fig. S6**A**, an indium (In) wire was used as the working electrode, and a single crystal of $NbSe_2$ was mechanically pressed onto it. A platinum (Pt) foil, chosen for its chemical inertness, served as the counter electrode.

For example, in the case of [(*S*)-MBMIm]$^+$ intercalation, a constant voltage of −1.2 V was applied to the [(*S*)-MBMIm][TFSI] ionic liquid at 100 °C under ambient atmosphere, after immersing the electrodes in the ionic liquid. Although effective for millimeter-scale crystals, setup A becomes challenging for very small samples (*e.g.*, ≈ 50 µm × 50 µm), which are required for high-quality synchrotron XRD measurements. To overcome this limitation, two alternative setups (B and C) were developed.

Setup B: In this configuration (Fig. S6**C**), a custom-built glass cell was used. A Pt foil attached to the bottom of a cylindrical glass tube served as the working electrode, onto which $NbSe_2$ powder was placed. The cell was filled with the ionic liquid, and a Pt counter electrode was immersed in the liquid, carefully positioned to avoid contact with the bottom electrode. To prevent electrical shorting, a BEMCOT™ Clean Wipe-P sheet was inserted between the two electrodes as a spacer.

Setup C: In this configuration (Figs. S6**E** and **F**), a coin-cell-type apparatus was employed for intercalation. The cell consists of Pt electrodes and a separator soaked in ionic liquid. Intercalation performed under identical voltage and temperature conditions yielded uniformly intercalated samples, confirming the reproducibility and homogeneity of this method.

ARPES measurement of (*S*)-MBMIm/$NbSe_2$

To evaluate the effects of molecular intercalation on the electronic structure, we performed angle-resolved photoemission spectroscopy (ARPES) measurements on both pristine $NbSe_2$ and (*S*)-MBMIm/$NbSe_2$. The results are summarized in Fig. S7.

For pristine $NbSe_2$, the experimentally obtained band dispersion is in good agreement with the density functional theory (DFT) calculations, confirming the validity of the measurement setup. In contrast, for (*S*)-MBMIm/$NbSe_2$, although the momentum resolution was somewhat degraded due to sample quality, two key features were identified in the density of states (DOS). First, within the energy range –0.4 to 1 eV, a depletion of the DOS was observed, indicating the opening of a gap. In pristine $NbSe_2$, finite DOS exists in this range due to k_z dispersion. The disappearance of this dispersion upon intercalation implies that the electronic structure becomes effectively two-dimensional due to reduced interlayer coupling. This gap formation and loss of k_z dispersion are both reproduced by DFT calculations performed using an expanded interlayer spacing of 12.6 Å. Second, a noticeable electron doping effect is observed. Comparison between the experimental spectra and DFT calculations shows that the bands in (*S*)-MBMIm/$NbSe_2$ are shifted toward lower energies, consistent with electron doping. Since the DFT calculations consider only the expansion of interlayer spacing, this shift evidence that electrons are transferred from the cationic [(*S*)-MBMIm]$^+$ molecules to the $NbSe_2$ layers.

In conclusion, ARPES measurements on (*S*)-MBMIm/$NbSe_2$ reveal two major effects of molecular intercalation: (i) dimensional reduction of the electronic structure, and (ii) electron doping due to the intercalation of cationic molecules. These features are consistent with the ARPES results obtained for EMIm/$NbSe_2$(*17*), suggesting that such behavior is a general characteristic of cation-intercalated systems.

X-ray diffraction simulations for single-q lattice modulation

To elucidate the structural modulation responsible for the superstructure (SS) peaks, we performed X-ray diffraction (XRD) simulations. As discussed in the main text, the SS peaks are attributed to modulations within the $NbSe_2$ host layers. Accordingly, the intercalated molecules are omitted from the simulation model.

In this section, we first assume that the experimentally observed satellite peaks arise from three rotational domains of a single-q modulation (Fig. S8**A**). In-plane displacements of Nb and Se atoms within each $NbSe_2$ layer should be negligibly small because no satellite peaks are observed in the h k 0 plane (Fig. 1**J**). As described in the main text, the modulation period is a_{SS} = 32.7 Å. Considering lattice matching with molecular ordering (MO) periodicity a_{MO} = 6.29 Å, two commensurate candidates are $a_{SS} = 11a \sim 6a_{MO}$ = 37.74 Å or $a_{SS} = 9a \sim 5a_{MO}$ = 31.45 Å. In this study, we adopted the $a_{SS} = 11a$ as the superlattice constant for the simulations.

We compare the simulated results and corresponding experimental XRD patterns in the h k 13 plane (Fig. S8**B**) and in the h k 14 plane (Fig. S8**C**). As the model for the out-of-plane modulation (namely, out-of-plane displacements of Nb and Se atoms), we considered two sinusoidal-type atomic displacements: one with the in-phase relation (model shown in Fig. S8**D**, simulated results in Figs. S8**E** and S8**F**) and the other with the anti-phase relation between adjacent layers (model shown in Fig. S8**G**, simulated results in Figs. S8**H** and S8**I**). The displacement Δc for both Nb and Se atoms are defined as:

$$\Delta c = \pm \Delta \sin\left(\frac{2\pi}{11(\sqrt{3}a/2)} q\right)$$

where a represents the a-axis lattice parameter of pristine $NbSe_2$, and the sign depends on the phase relationship between layers. The modulation amplitude Δ was fixed at 0.05 % of the c-axis length (0.012 Å).

Crucially, comparing these models allows us to determine the relative phase between layers. The in-phase sinusoidal-type modulation fails to capture the key experimental feature, *i.e.*, satellite peaks around the forbidden (0,0,13) reflection. This is consistent with the fact that, for the in-phase modulation, satellites around (0,0,l) (l is odd) are eliminated by the extinction rule. In contrast, the anti-phase sinusoidal-type modulation successfully reproduces satellite pattern in the h k 13 plane. Therefore, we conclude that the adjacent layers modulate in the anti-phase or with a finite phase difference.

This type of out-of-plane lattice modulation stands in contrast to conventional charge density waves in transition metal dichalcogenides (*e.g.*, pristine 2H-$NbSe_2$), which typically induce in-plane atomic displacements(*59*). In contrast, it is a well-discussed phenomenon in the context of moiré superlattices, where it is often termed “atomic corrugation”(*60–63*). In twisted bilayer graphene, for instance, first-principles calculations predict that the moiré potential induces an out-of-plane corrugation with an amplitude of 0.08-0.12 Å(*64*). Our simulations reveal that a similar type of lattice modulation occurs in the CSS phase. We attribute this modulation to a synergistic effect of several coupled degrees of freedom, which include the ionic host-guest interaction, local lattice relaxations, and even the packing constraints of the molecular sublattice.

Regarding the c-glide symmetry, the (h,h,l) (l is odd) peaks were clearly observed in the experimental results, corresponding to the broken c-glide symmetry in the CSS phase. However, in our simulations, these peaks were either absent or several orders of magnitude smaller than the experimentally observed (h,h,l) (l is odd) peaks. This suggests that the experimentally observed c-glide symmetry breaking is associated with additional types of structural modulation beyond those included in our simplified model.

For comparison, we also examined breathing-type modulations. The model with the in-phase relation between adjacent layers is shown in Fig. S8**J**, and the corresponding simulated patterns are shown in Figs. S8**K** and S8**L**. The model with the anti-phase relation between adjacent layers is shown in Fig. S8**M**, with the corresponding simulations in Figs. S8**N** and S8**O**. We found that the breathing-type models with in-phase and anti-phase modulations yield nearly identical results to the sinusoidal-type in-phase and anti-phase, respectively. This consistency strongly suggests that the anti-phase relationship between layers is the dominant factor governing the XRD pattern.

X-ray diffraction simulations for triple-*q* lattice modulation

In previous section, we considered a model where $NbSe_2$ layers are modulated in a single-*q* direction with three rotational domains. Here, we show that XRD simulations based on a triple-*q* modulation are also compatible with corresponding experimental XRD patterns in the *h k* 13 plane (Fig. S8**B**) and in the *h k* 14 plane (Fig. S8**C**). As shown in Fig. S9**A**, plotted with the same parameters as the anti-phase sinusoidal-type modulation (Figs. S8**G**), similar SS peaks are obtained for the triple-*q* modulation. Although additional satellite peaks appear in the simulation, their intensities become negligible as the modulation amplitude is reduced (Fig. S9**B**). However, as in the single-*q* modulation case, this model fails to reproduce the intense (*h,h,l*) (*l* odd) peaks observed experimentally. This result suggests that additional types of structural modulation to break *c*-glide symmetry are present.

In conclusion, the experimentally observed SS pattern is consistent with either the single-*q* modulation with three rotational domain or the triple-*q* modulation. Discriminating between these possibilities will require local probes such as transmission electron microscopy (TEM) or scanning tunneling microscopy (STM), which we leave for future work.

Structural analysis of the high-temperature phase

In the high-temperature regime, where the intercalated molecules do not exhibit long-range order, the crystal structure is expected to retain the symmetry of pristine $NbSe_2$ while accommodating an expanded interlayer distance. Here, we analyze the crystal structure under this assumption by modeling only the $NbSe_2$ framework and neglecting possible diffuse scattering or weak modulations originating from disordered molecules or short-range molecular correlations.

The refined structural parameters are summarized in Supplementary Tables S3 and S4. The Nb–Se bond length in this phase is determined to be 2.601(1) Å, which is essentially unchanged from that of pristine $NbSe_2$. This indicates that the local coordination environment within each $NbSe_2$ layer remains intact despite the substantial expansion of the van der Waals gap. In other words, intercalation primarily modifies the interlayer spacing without significantly perturbing the intralayer $NbSe_2$ structure.

Space group analysis of the CSS phase

Here we discuss the symmetry of the crystal structure in the CSS phase. The threefold rotational symmetry is expected to be preserved if the superstructure adopts the triple-q modulation, whereas it should be broken in the single-q configuration.

We first consider the case with the triple-q modulation. Starting from the space group of $P6_3/mmc$ in the high-temperature phase, we checked all subgroups while retaining the threefold rotational axis. The extinction rules provide two additional constraints: the appearance of (h,h,l) (l is odd) reflections demonstrates the broken c-glide symmetry, whereas the absence of $(0,0,l)$ (l is odd) reflections demonstrates that the 6_3 screw axis is preserved. The space groups consistent with these conditions are summarized in Fig. S10, from which the plausible candidates are $P6_322$, $P6_3m$, or their subgroups. In the single-q case, the threefold rotational symmetry assumed for the triple-q scenario is additionally broken. Therefore, the symmetry is reduced from $P6_322$ in the high-temperature phase to $Cmcm$, and further reduced by breaking the c-glide symmetry while preserving the 2_1 screw axis. The corresponding candidate space groups for the single-q case are summarized in Fig. S11. Such symmetry determination is critical, as the lack of inversion symmetry in chiral $P6_322$ will lead to qualitative differences in physical properties such as nonreciprocal transport and optical activity, compared to centrosymmetric $P6_3/m$. Therefore, complementary techniques like second harmonic generation (SHG) or local structural probes are essential to unambiguously distinguish among these candidates.

Comparison of the CSS phase with other molecular intercalants

To investigate the molecular species dependence of the cooperative superstructure (CSS) phase, synchrotron XRD measurements were also performed on $NbSe_2$ intercalated with other cations, namely, 1-Butyl-3-methylimidazolium ($[BMIm]^+$) and 1-Ethyl-3-methylimidazolium ($[EMIm]^+$). As shown in Fig. S1, both systems exhibit successful intercalation throughout the bulk crystal, as confirmed by the systematic shifts of the pristine $NbSe_2$ reflections and by an increase in the *c*-axis lattice parameter. These results reproduce the findings of previous study(*17*). Synchrotron XRD measurements for these samples are presented in Fig. S12.

In EMIm/$NbSe_2$, no signatures associated with the CSS phase were detected: neither molecular-ordering (MO) peaks, superstructure (SS) peaks, nor additional Bragg peaks related to symmetry breaking were observed. This indicates that the intercalated EMIm molecules remain in a disordered liquid- or glass-like state without crystallization. This conclusion is consistent with previous reports(*17*), in which no resistivity anomalies suggesting molecular ordering were observed up to 300 K. In contrast, BMIm/$NbSe_2$ exhibited distinct MO peaks, demonstrating that the BMIm molecules undergo crystallization within the van der Waals gaps. Compared with the (*S*)-MBMIm/$NbSe_2$ system, the ordering periodicity of BMIm/$NbSe_2$ differs, with a molecular-ordering wavevector of $q_{\mathrm{MO}} = 2/3\ a^*$, corresponding to a commensurate relationship with the $NbSe_2$ lattice. However, no SS peaks originating from modulation of the $NbSe_2$ layers were observed, suggesting that molecular shape and packing geometry play crucial roles in determining whether molecular ordering couples cooperatively to the host lattice to form the CSS phase.

All three cations [(*S*)-$MBMIm^+$, $BMIm^+$, and $EMIm^+$] share the same imidazolium backbone, differing only in the length and chirality of their alkyl side chains (Fig. S1**A**). Nevertheless, they exhibit qualitatively distinct behaviors in relation to the CSS: (*S*)-$MBMIm^+$ induces both molecular and host-lattice ordering, $BMIm^+$ shows only molecular ordering, and $EMIm^+$ remains disordered. This systematic variation underscores that subtle differences in molecular structure profoundly affect the cooperative coupling between molecular and lattice degrees of freedom. These findings highlight the rich tunability of CSS formation and provide a design guideline for molecular-level control in hybrid superlattice materials.

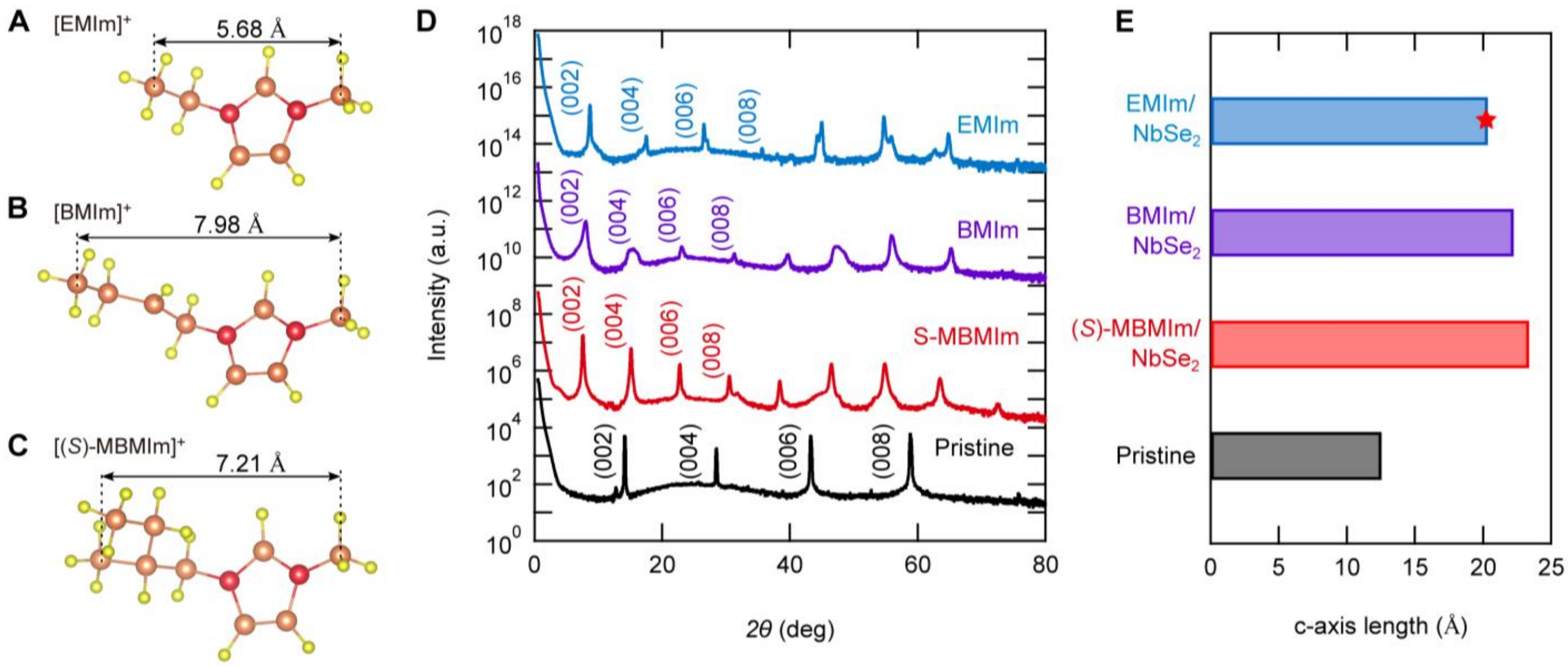

Figure S1 | X-ray diffractions and *c*-axis lengths of $NbSe_2$ with several molecular-cation intercalants

(**A** to **C**), Schematic drawing of the molecular structures of $[EMIm]^+$ (1-Ethyl-2,3-dimethyl-imidazolium) (**A**), $[BMIm]^+$ (1-Butyl-2,3-dimethyl-imidazolium) (**B**), and $[(S)\text{-}MBMIm]^+$ ((*S*)-1-(2-Methylbutyl)-3-methylimidazolium) (**C**). The lengths shown in the figures are defined as the distance between the end carbon atoms. (**D**), Room-temperature X-ray diffraction (XRD) patterns of pristine (unintercalated) $NbSe_2$ and $NbSe_2$ intercalants of $[(S)\text{-}MBMIm]^+$, $[BMIm]^+$, and $[EMIm]^+$. (**E**), Comparison of *c*-axis lengths estimated from XRD. The red star shows the *c*-axis length of previous reported EMIm/$NbSe_2$(*17*), which is consistent with our samples.

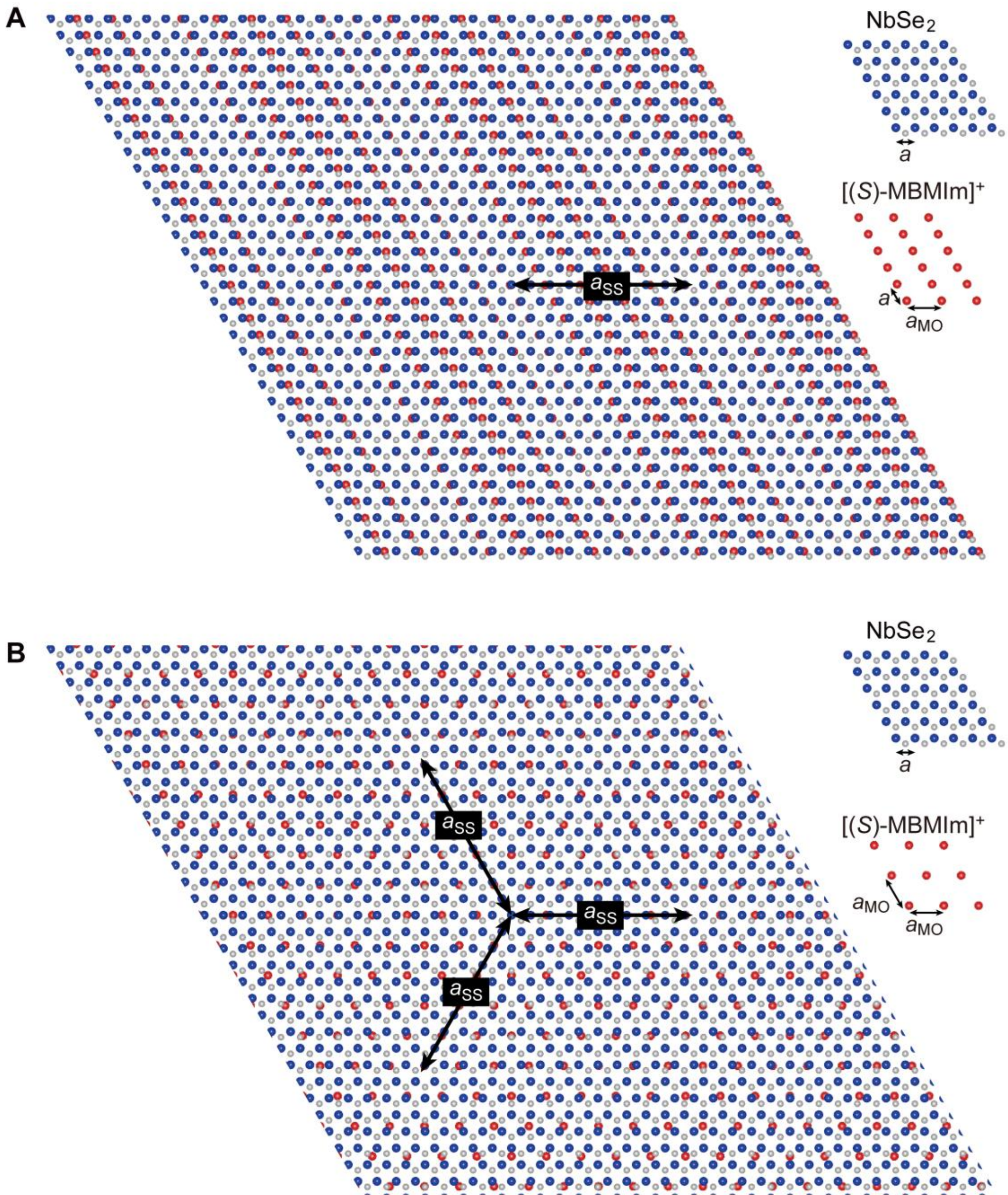

Figure S2 | Moiré superlattices formed by stacking $NbSe_2$ and ordered organic molecules. (**A** and **B**), Real-space moiré patterns simulated by stacking a single layer of $NbSe_2$ with a layer of organic molecules ordered in a single-*q* configuration (**A**) and a triple-*q* configuration (**B**). Nb and Se atoms in $NbSe_2$ are represented by blue and grey circles, respectively, while each organic molecule is depicted schematically as a single red circle. The lattice constants of $NbSe_2$, the ordered molecular layer, and the long-period superlattice are taken from experiment as a = 3.46 Å, a_{MO} = 6.29 Å, and a_{SS} = 32.7 Å, respectively.

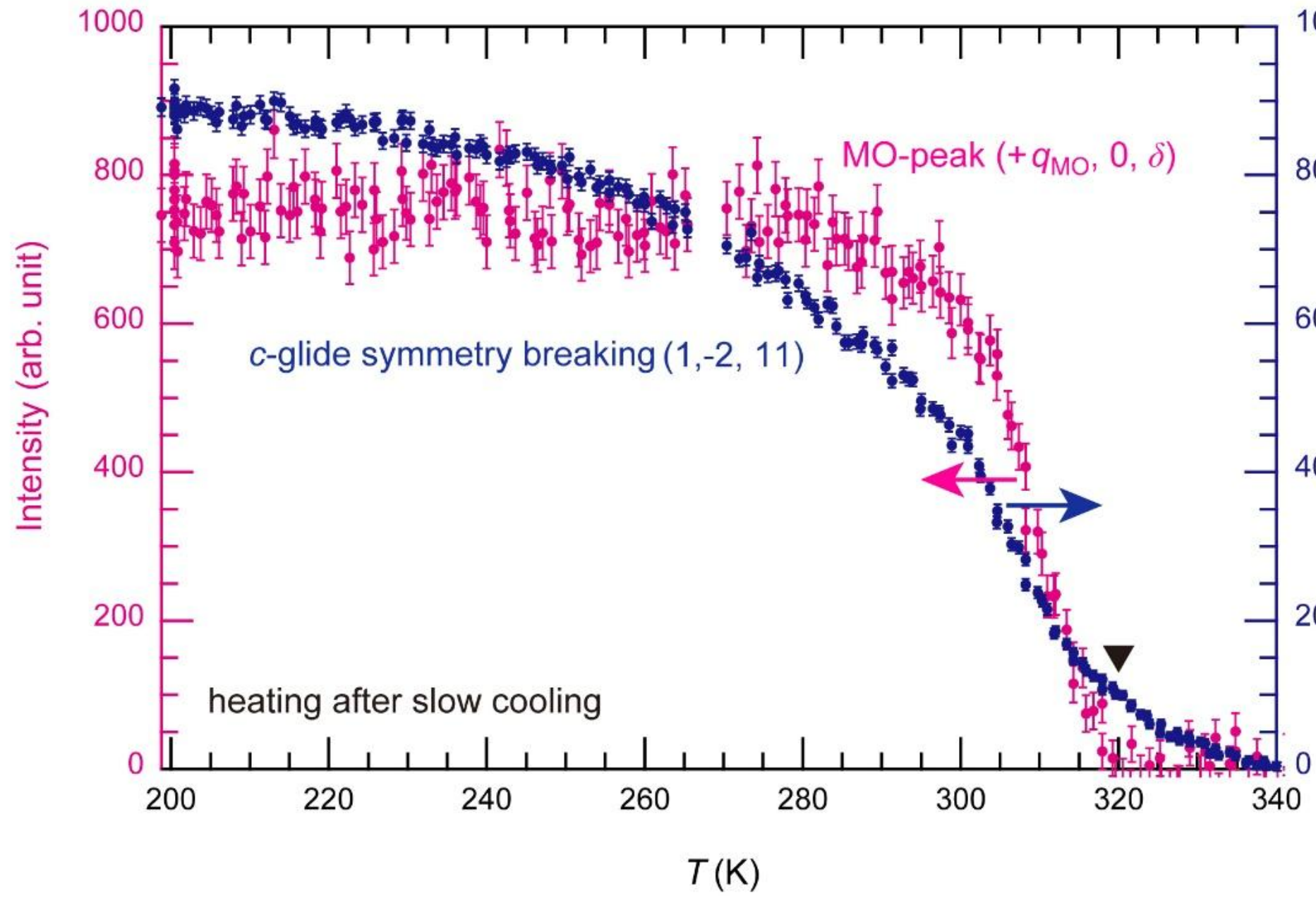

Figure S3 | Temperature dependence of diffraction peaks in (*S*)-MBMIm/NbSe$_2$
Temperature dependence of the diffraction intensities of the MO peak (+q_{MO}, 0, 1.1) and the (1,–2,11) peak during heating after slow cooling. The (1,–2,11) reflection, which is symmetry-equivalent to the (1,1,11) reflection by the C_3 rotational symmetry, belongs to the (h,h,l) (l is odd) reflections that are forbidden by the c-glide symmetry; therefore, its disappearance at high temperature corresponds to the restoration of the c-glide symmetry. Both peaks disappear simultaneously at the melting temperature T_m as indicated by the black triangle, demonstrating that the breaking of the c-glide symmetry is intrinsically associated to the formation of the CSS phase.

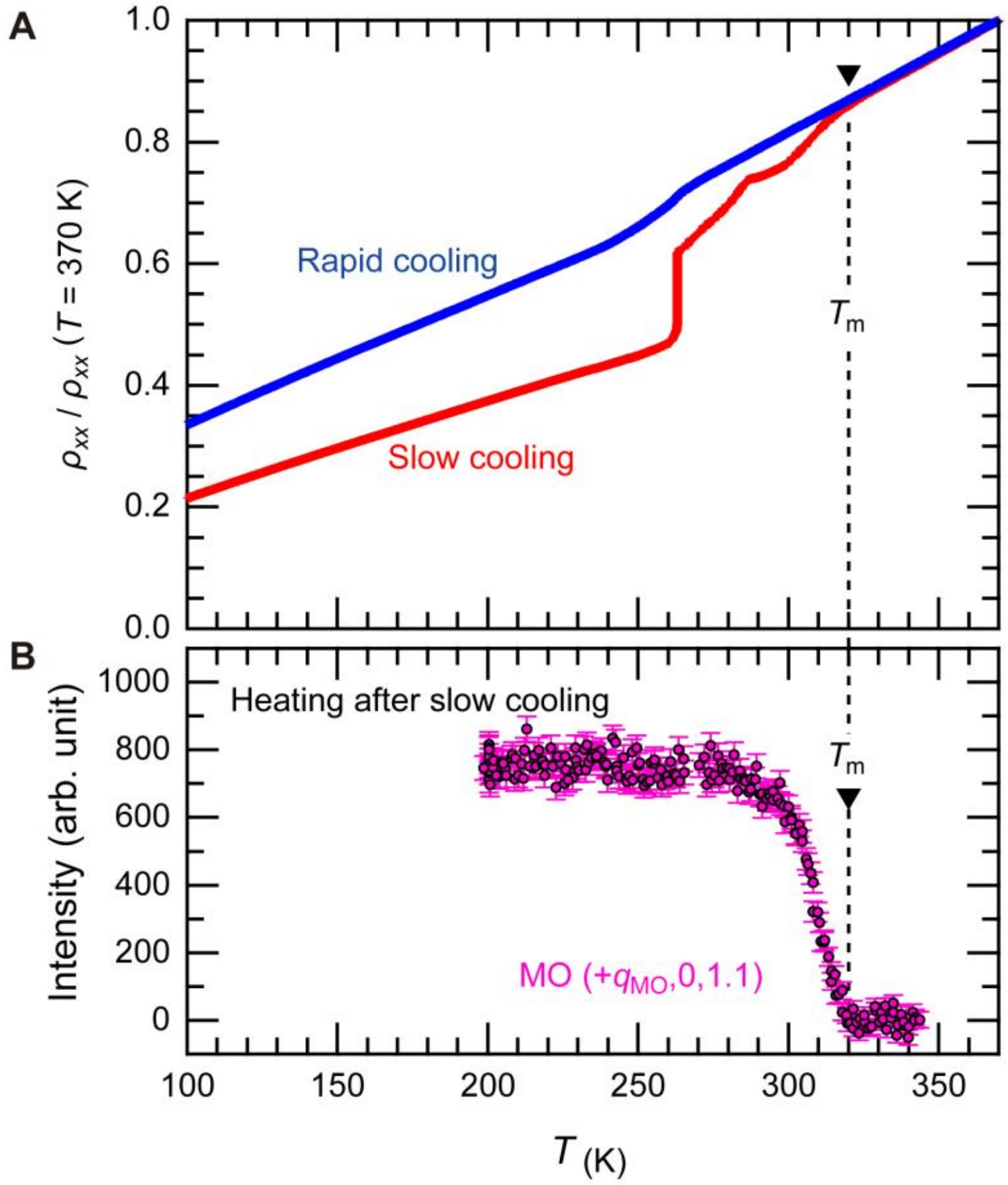

Figure S4 | Correspondence between the CSS transition and resistivity anomaly in (*S*)-MBMIm/$NbSe_2$.

(**A**), Temperature dependence of the normalized resistivity of (*S*)-MBMIm/$NbSe_2$ measured under slow cooling (2 K min^{-1}, red) and rapid cooling (11 K min^{-1}, blue). The gray curve represents $d\rho_{xx}/dT$ under slow cooling. The onset of deviation between the two cooling curves, corresponding to the upper bound of the transition temperature T_m = 320 K. (**B**), Temperature dependence of the diffraction intensities of the molecular-ordering (MO) peaks measured on the same sample as in **A**. The disappearance of the MO peaks upon heating defines the melting temperature T_m, which closely matches the resistivity anomaly.

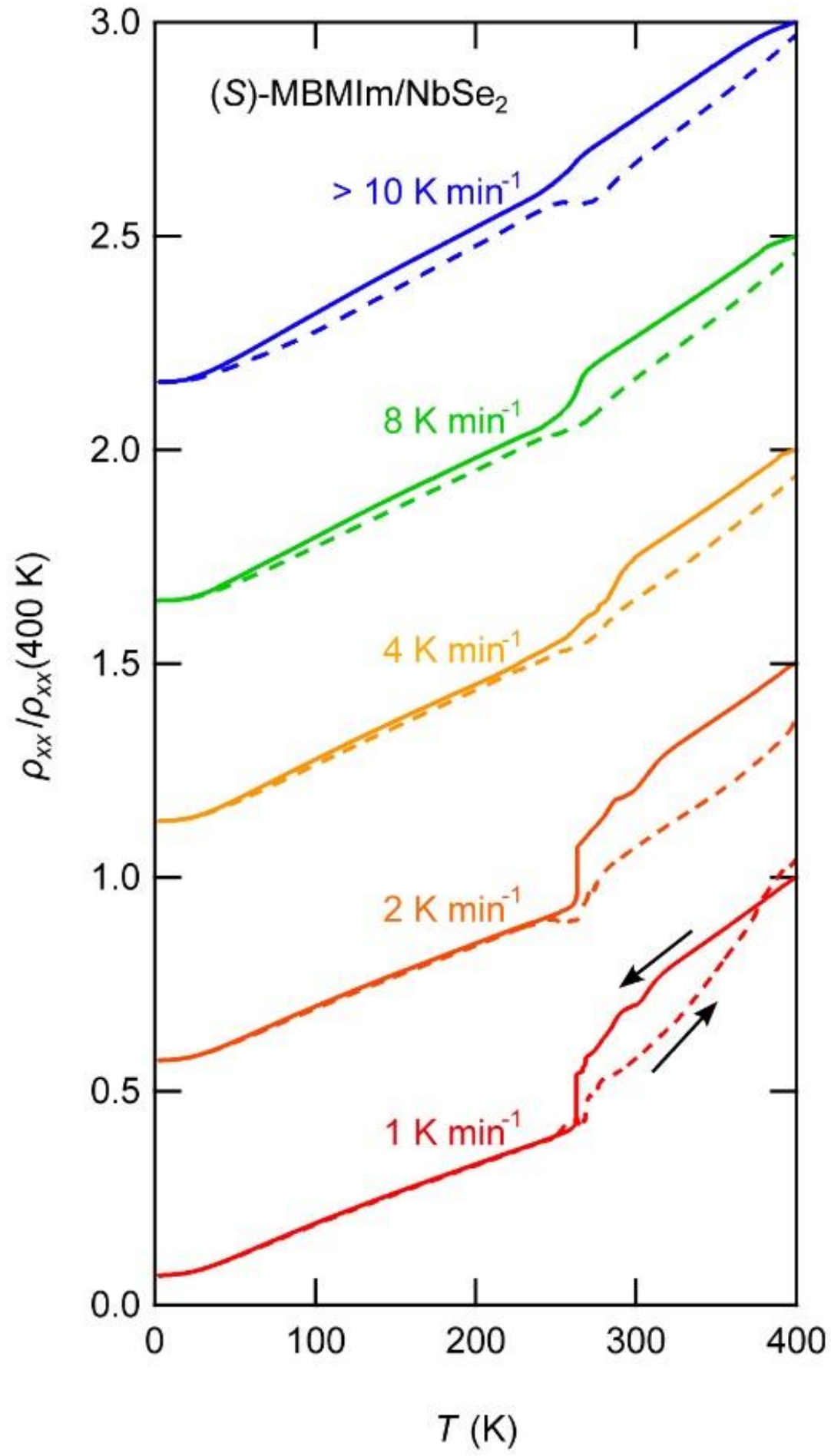

Figure S5 | Temperature dependence of resistivity with varying sweep rate of temperature. Temperature dependence of resistance of (*S*)-MBMIm/$NbSe_2$ measured under various sweep rates. Solid lines denote the cooling process, and dashed lines denote the heating process, as indicated by the arrows.

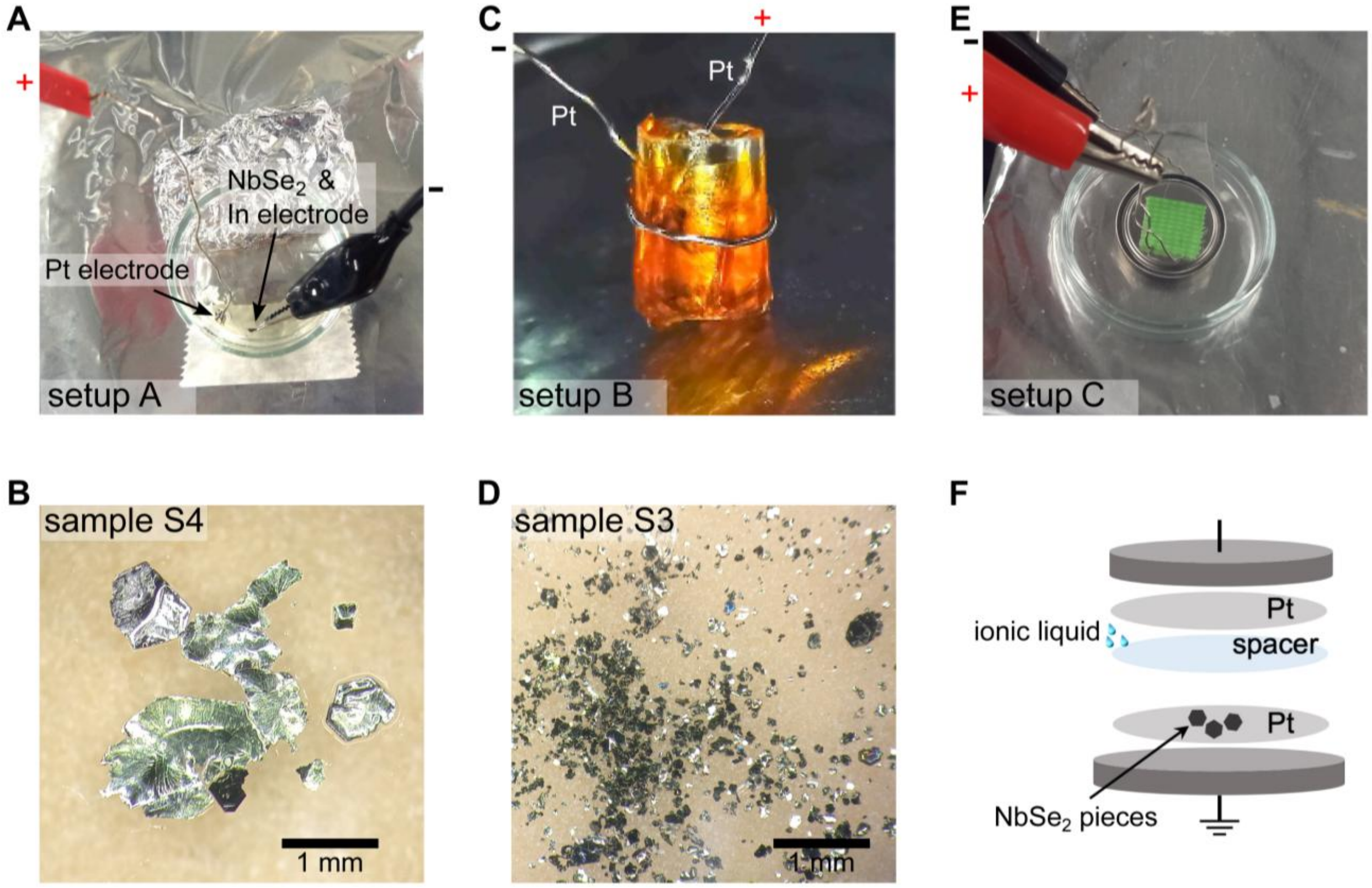

Figure S6 | Experimental setup for intercalation and photographs of samples
(**A**), Photograph of setup A. (**B**), Photograph of sample S4, prepared using setup A. (**C**), Photograph of setup B. (**D**), Photograph of sample S3, prepared using setup B. (**E**), Photograph of setup C, which consists of a coin-cell case, Pt electrodes, and a spacer soaked in ionic liquid. (**F**), Schematic illustration of setup C.

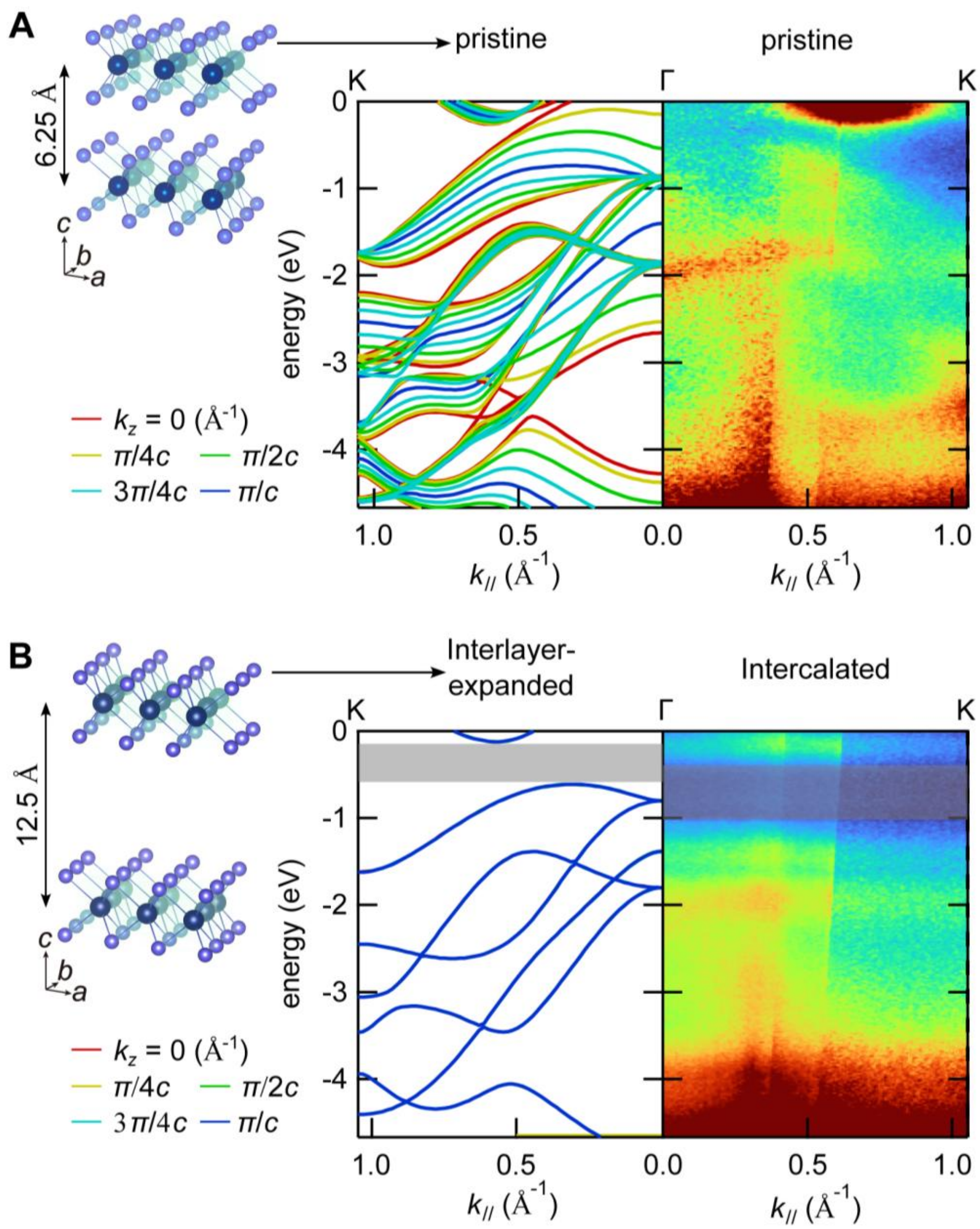

Figure S7 | Electronic structures of pristine and intercalated $NbSe_2$.
(**A**), Comparison of the band structures of pristine $NbSe_2$ calculated by DFT and measured by ARPES along the Γ–K direction. Left panels show the crystal structures used for the DFT calculations. (**B**), Comparison of the band structures of (*S*)-MBMIm/$NbSe_2$ calculated by DFT and measured by ARPES along the Γ–K direction. Left panels show the crystal structures used for the DFT calculations. Note that the DFT calculations were performed without intercalated molecules but with an interlayer spacing of 12.6 Å. The gray regions highlight the band gap.

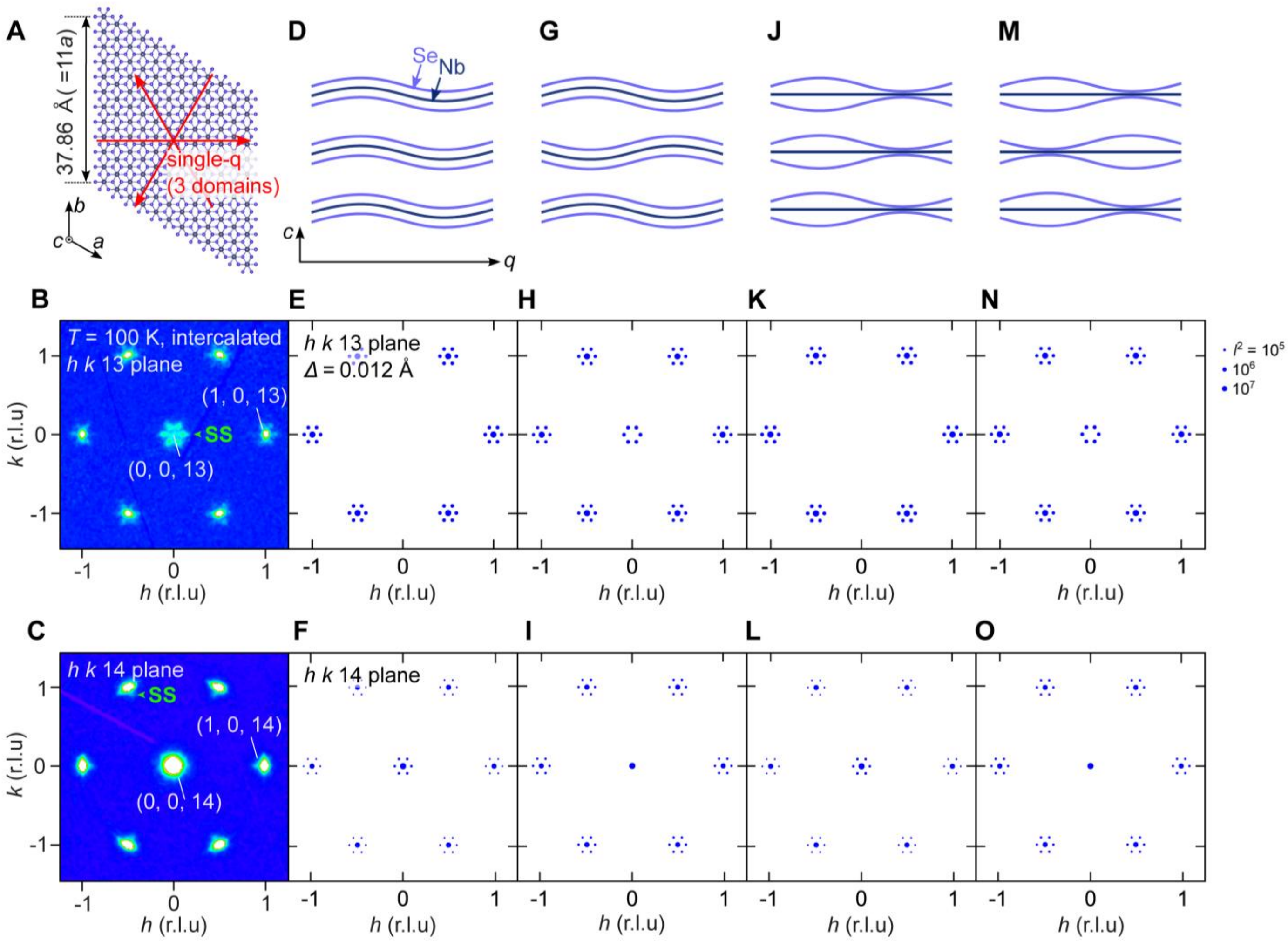

Figure S8 | Simulations of X-ray diffraction patterns for single-*q* modulations

(**A**), Schematic of the single-*q* modulations used for the X-ray diffraction (XRD) simulations in the CSS phase of (*S*)-MBMIm/$NbSe_2$. An $11a \times 11b$ supercell is constructed based on the original crystal structure. (**B** and **C**), Experimental XRD patterns in the *h k* 13 plane (**B**) and in the *h k* 14 plane (**C**). (**D**), Schematic of sinusoidal-type modulation, $\Delta \sin\left(\frac{2\pi}{11(\sqrt{3}a/2)}q\right)$, applied to both Se and Nb atoms with the in-phase relation between adjacent layers. Here, q represents the modulation vector and a represents the *a*-axis lattice parameter. (**E** and **F**), Simulated reciprocal maps in the *h k* 13 plane (**E**) and in the *h k* 14 plane (**F**) for the configuration shown in **D**. (**G**), Schematic of sinusoidal-type modulation with the anti-phase relation between adjacent layers. (**H** and **I**), Simulated reciprocal maps in the *h k* 13 plane (**H**) and in the *h k* 14 plane (**I**) for the configuration shown in **G**. (**J**), Schematic of breathing-type modulation, $\Delta \sin\left(\frac{2\pi}{11(\sqrt{3}a/2)}q\right)$, applied only to the Se atoms with the in-phase relation between adjacent layers. Nb atoms are fixed at their original positions. (**K** and **L**), Simulated reciprocal maps in the *h k* 13 plane (**K**) and in the *h k* 14 plane (**L**) for the configuration shown in **J.** (**M**), Schematic of breathing-type modulation with the anti-phase relation between adjacent layers. (**N** and **O**), Simulated reciprocal maps in the *h k* 13 plane (**N**) and in the *h k* 14 plane (**O**) for the configuration shown in **M.** The amplitude Δ is

fixed at 0.012 Å, and peaks with intensities below 10^1 are omitted in all XRD simulations. Intercalated molecules between layers are not considered in the models.

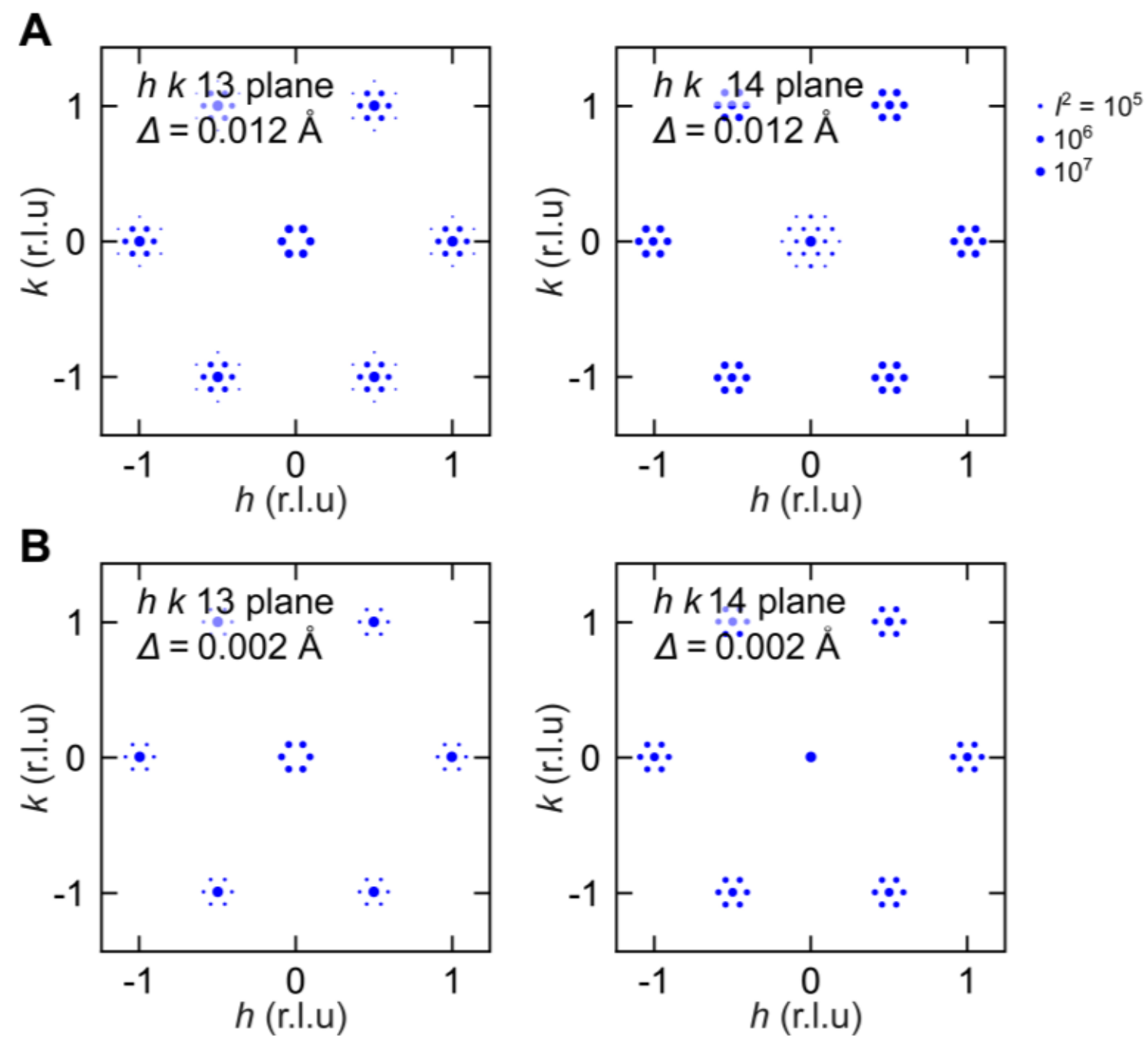

Figure S9 | Simulations of X-ray diffraction patterns for triple-*q* modulations
(**A** and **B**), Simulated reciprocal maps in the h k 13 plane and in the h k 14 plane for triple-q modulations with $11a \times 11b$ periodicity. The modulation amplitude Δ is fixed at 0.012 Å (**A**) and 0.002 Å (**B**). All other simulation parameters are identical to those used in Fig. S3**G**. Peaks with intensities below 10^1 are omitted.

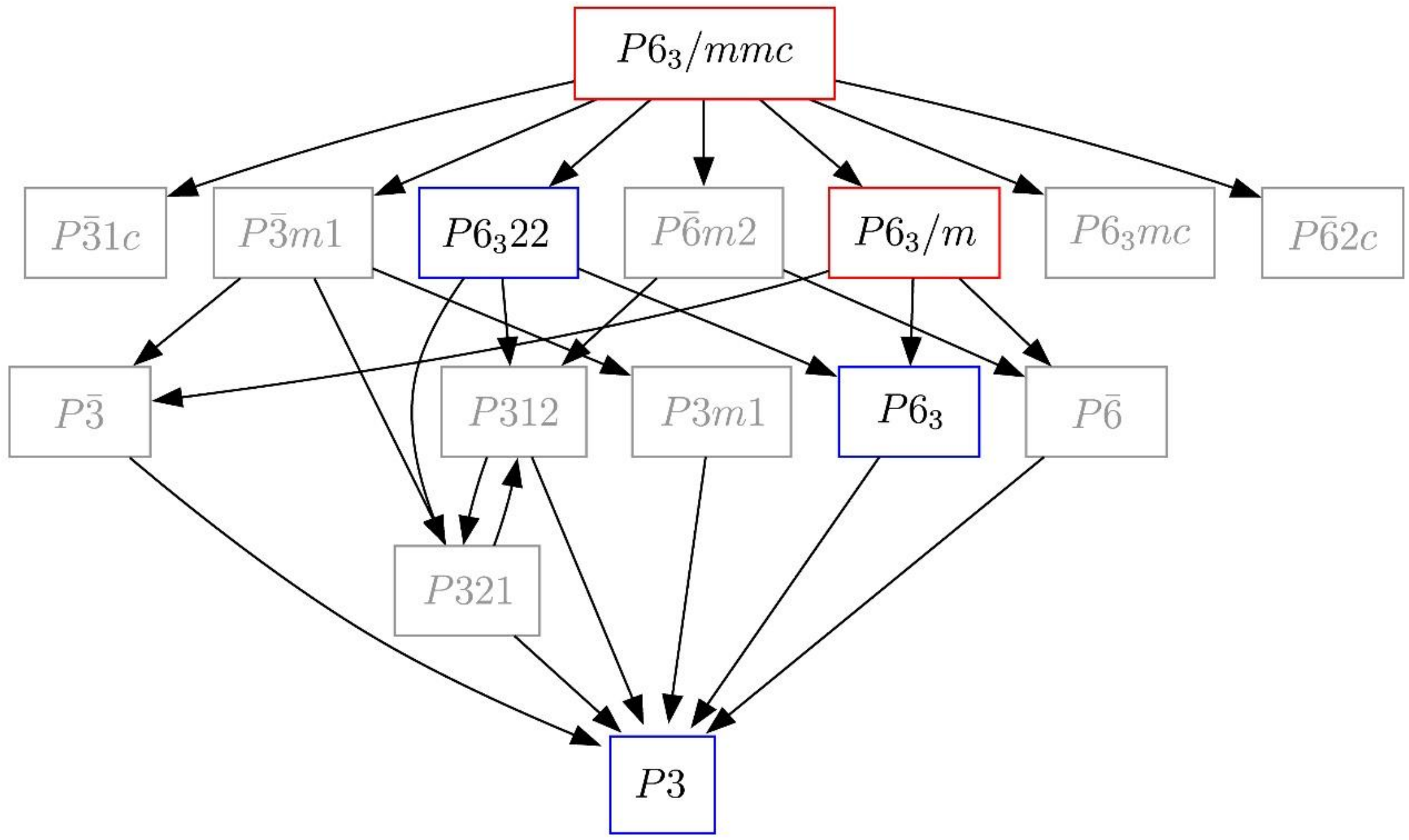

Figure S10 | Candidate space groups for the CSS phase with triple-*q* modulations.
List of possible space groups for the CSS phase obtained from subgroup analysis of $P6_3/mmc$, assuming preservation of the threefold rotational axis with triple-q modulation. Space groups shown in gray retain the c-glide symmetry or break 6_3 screw rotation symmetry and can therefore be excluded based on the observed extinction-rule. Among the remaining candidates, those enclosed by red boxes are centrosymmetric, whereas those enclosed by blue boxes are noncentrosymmetric. Arrows indicate group-subgroup relationships between the corresponding space groups.

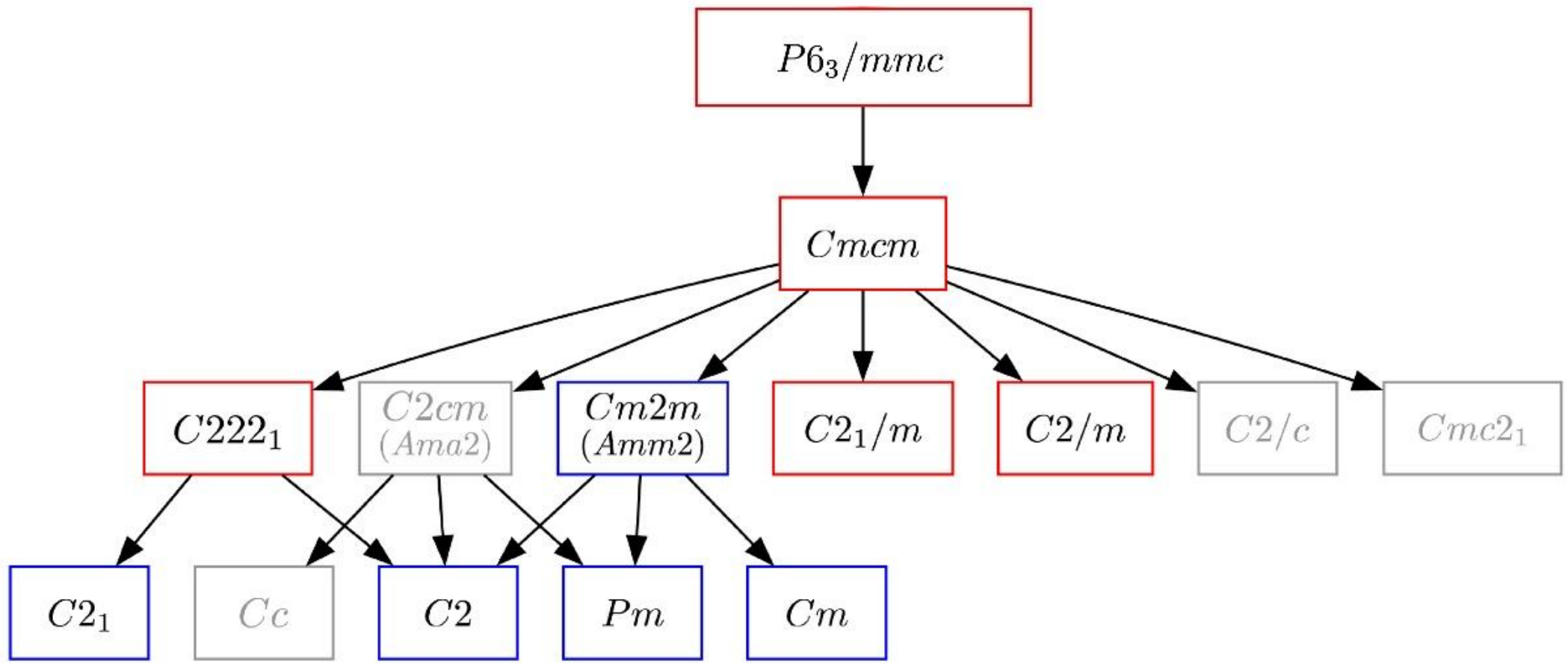

Figure S11 | Candidate space groups for the CSS phase with single-*q* modulations.
List of possible space groups for the CSS phase obtained from subgroup analysis of $P6_3/mmc$ under the assumption that the threefold rotation axis is broken by a single-q modulation. Space groups shown in gray retain the c-glide symmetry or break 2_1 screw rotation symmetry and can therefore be excluded based on the observed extinction-rule. Among the remaining candidates, those enclosed by red boxes are centrosymmetric, whereas those enclosed by blue boxes are noncentrosymmetric. Arrows indicate group–subgroup relationships between the corresponding space groups.

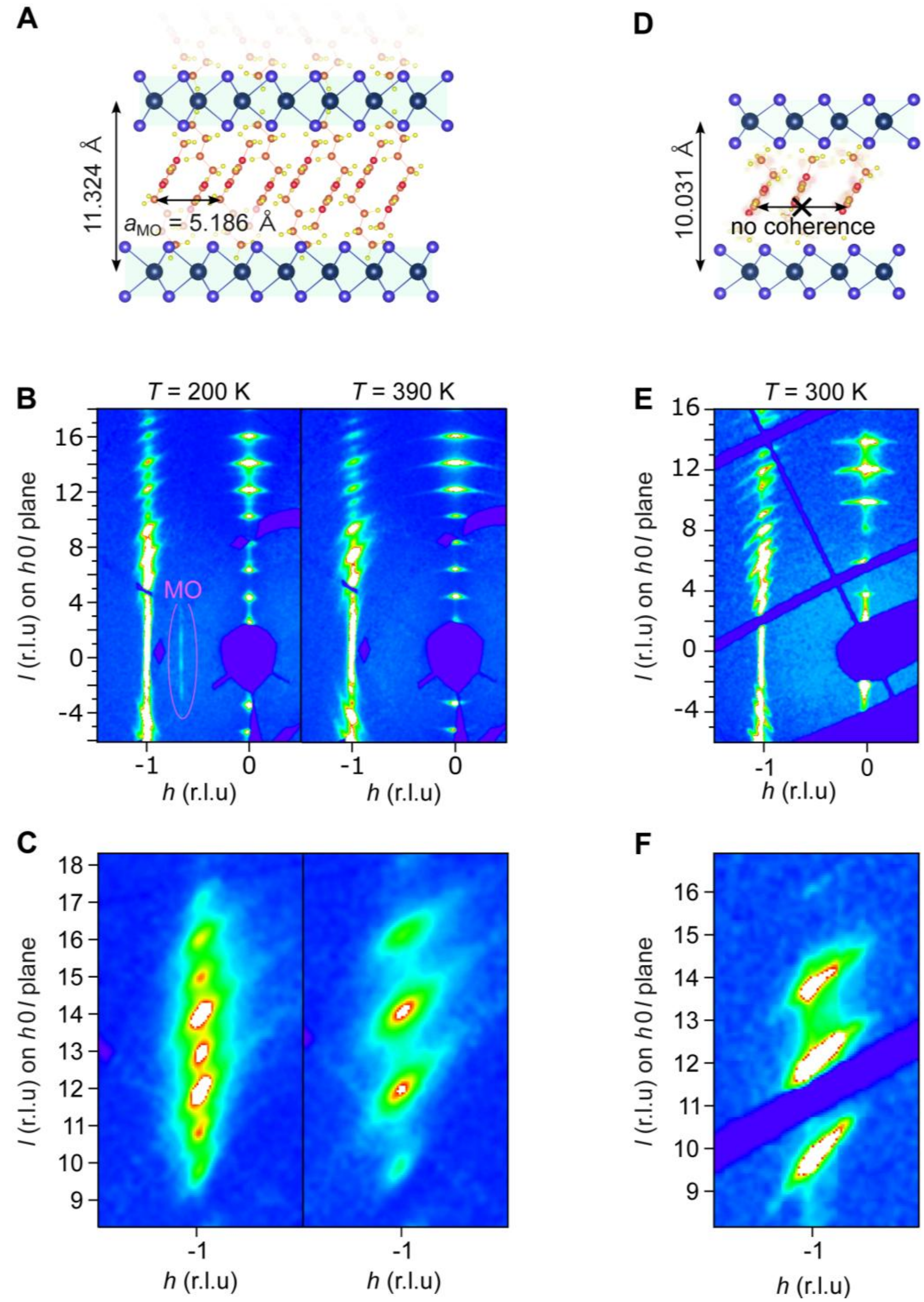

Figure S12 | Synchrotron X-ray diffraction for other cation-intercalated $NbSe_2$.
(**A**), Schematic illustration of BMIm/$NbSe_2$. The interlayer spacing and molecular-ordering (MO) periodicity a_{MO} were obtained from results of X-ray diffraction (XRD).
(**B**), Synchrotron XRD reciprocal-space maps of BMIm/$NbSe_2$ in the h 0 l plane at 200 K and 390 K. At 200 K, MO peaks are observed at $q_{MO} \approx 2/3\ a^*$, which disappear completely at 390 K. These peaks correspond to a molecular-ordering periodicity of $a_{MO} \approx 3/2\ a$ = 5.186 Å. The slightly stronger intensity of the MO peaks of $l = 2n + 1$ than that of $l = 2n$ suggests weak interlayer coherence, with alternating molecular orientations between adjacent layers. The

melting temperature T_m, where the MO peaks vanish, is estimated to be 380-390 K. In contrast to (*S*)-MBMIm/$NbSe_2$, no superstructure (SS) peaks arising from modulation of the $NbSe_2$ layers are detected, implying that geometrical details of molecular structure plays a crucial role in the formation of the CSS phase. (**C**), Synchrotron XRD reciprocal-space maps of BMIm/$NbSe_2$ in the *h h l* plane at 200 K and 390 K. Similar to (*S*)-MBMIm/$NbSe_2$, peaks at $l = 2n + 1$, indicative of *c*-glide symmetry breaking, are visible at 200 K but vanish at 390 K, signifying recovery of the original $NbSe_2$ symmetry. (**D**), Schematic illustration of EMIm/$NbSe_2$. The interlayer spacing was obtained from results of XRD. (**E**), Synchrotron XRD reciprocal-space map of EMIm/$NbSe_2$ in the (h,0,l) plane at 300 K. Although *c*-axis expansion is evident, no MO or SS peaks are observed, indicating the absence of coherent molecular ordering. (**F**), Synchrotron XRD reciprocal-space map of EMIm/$NbSe_2$ in the *h h l* plane at 300 K. The absence of reflections at $l = 2n + 1$ confirms that the system retains the same *c*-glide symmetry as pristine $NbSe_2$.

Supplementary Table S1 | Sample information of (*S*)-MBMIm/$NbSe_2$

sample no.	intercalation setup	Used in which figures	size	T_m	comment
S1	B	Fig. 1D	1 mm× 1 mm× 16 μm	-	for Raman
S2	B	Fig. 1H-1J, 2	50 um× 50 um× 16 μm	~ 310 K	for XRD
S3	B	Fig. 3B (XRD)	50 um× 50 um× 16 μm	-	for XRD with quenching
S4	A	Fig. 1C, 3B (transport), 4A-B, S1D, S4, S5	1 mm× 1 mm× 16 μm	~ 320 K	for XRD and electrical transport
S5	A	Fig. S7B	0.5 mm× 0.5 mm× 16 μm	-	for ARPES
S6	C	-	1 mm× 1 mm× 16 μm	-	To confirm the validity of setup 1C

Supplementary Table S2 | Sample information of $NbSe_2$ intercalated with other molecules

sample no.	intercalation method	Used in which figures	size	comment
B1	C	Fig. S1D, S12B-C	1 mm× 1 mm× 32 μm	BMIm intercalated
E1	C	Fig. S1D, S12E-F	1 mm× 1 mm× 32 μm	EMIm intercalated

Supplementary Table S3 | Structural parameters obtained from structural refinement on the high-temperature phase.

Atom	Wyckoff position	Site symmetry	x	y	z
Nb1	2c	-6m2	0.33333	0.66667	0.25000
Se1	4f	3m.	0.66667	0.33333	0.17655(7)

Atom	U_{11} (Å^2)	U_{22} (Å^2)	U_{33} (Å^2)	U_{12} (Å^2)	U_{13} (Å^2)	U_{23} (Å^2)
Nb1	0.0091(4)	$=U_{11}$	0.044(1)	0.0045(2)	0	0
Se1	0.0094(4)	$=U_{11}$	0.044(1)	0.0047(2)	0	0

Supplementary Table S4 | Summary of crystallographic data of (*S*)-MBMIm/$NbSe_2$ in the high temperature phase.

Wavelength (Å)	0.352883
Crystal dimension (μm^3)	50*50*16
a (=b) (Å)	3.4632(2)
c (Å)	22.666(1)
α	90
γ	120
V (Å^3)	235.43(2)
Z	2
$(\sin\theta/\lambda)_{max}$ (Å^{-1})	1.05
N_{total}	487
R_1 (I>3σ / all)	10.44% / 11.26%
wR_2 (I>3σ / all)	15.48% / 15.56%
GOF (I>3σ / all)	9.58 / 8.82

Supplementary Table S5 | Summary of crystallographic data of (*S*)-MBMIm/$NbSe_2$ in the CSS phase.

Wavelength (Å)	0.352883
Crystal dimension (μm^3)	50*50*16
a (=*b*) (Å)	3.4621(2)
c (Å)	22.113(1)
α	90
γ	120
V (Å^3)	229.54(2)
Z	2
$(\sin\theta/\lambda)_{max}$ (Å^{-1})	1.06
N_{total}	6479

REFERENCES

1. J. Mannhart, D. G. Schlom, Oxide interfaces—An opportunity for electronics. *Science* **327**, 1607–1611 (2010).

2. H. Y. Hwang, Y. Iwasa, M. Kawasaki, B. Keimer, N. Nagaosa, Y. Tokura, Emergent phenomena at oxide interfaces. *Nat. Mater.* **11**, 103–113 (2012).

3. A. I. Buzdin, Proximity effects in superconductor-ferromagnet heterostructures. *Rev. Mod. Phys.* **77**, 935–976 (2005).

4. Y. Tokura, K. Yasuda, A. Tsukazaki, Magnetic topological insulators. *Nat. Rev. Phys.* **1**, 126–143 (2019).

5. E. Y. Andrei, D. K. Efetov, P. Jarillo-Herrero, A. H. MacDonald, K. F. Mak, T. Senthil, E. Tutuc, A. Yazdani, A. F. Young, The marvels of moiré materials. *Nat. Rev. Mater.* **6**, 201–206 (2021).

6. P. C. Adak, S. Sinha, A. Agarwal, M. M. Deshmukh, Tunable moiré materials for probing Berry physics and topology. *Nat. Rev. Mater.* **9**, 481–498 (2024).

7. L. Ju, A. H. MacDonald, K. F. Mak, J. Shan, X. Xu, The fractional quantum anomalous Hall effect. *Nat. Rev. Mater.* **9**, 455–459 (2024).

8. F. R. Gamble, F. J. Disalvo, R. A. Klemm, T. H. Geballe, Superconductivity in layered structure organometallic crystals. *Science* **168**, 568–570 (1970).

9. F. R. Gamble, J. H. Osiecki, M. Cais, R. Pisharody, F. J. Disalvo, T. H. Geballe, Intercalation complexes of lewis bases and layered sulfides: A large class of new superconductors. *Science* **174**, 493–497 (1971).

10. Y. Matsuo, K. Tahara, Y. Sugie, Synthesis of poly(ethylene oxide)-intercalated graphite oxide. *Carbon* **34**, 672–674 (1996).

11. Z. Li, Y. Zhao, K. Mu, H. Shan, Y. Guo, J. Wu, Y. Su, Q. Wu, Z. Sun, A. Zhao, X. Cui, C. Wu, Y. Xie, Molecule-confined engineering toward superconductivity and ferromagnetism in two-dimensional superlattice. *J. Am. Chem. Soc.* **139**, 16398–16404 (2017).

12. C. Wang, Q. He, U. Halim, Y. Liu, E. Zhu, Z. Lin, H. Xiao, X. Duan, Z. Feng, R. Cheng, N. O. Weiss, G. Ye, Y.-C. Huang, H. Wu, H.-C. Cheng, I. Shakir, L. Liao, X. Chen, W. A. Goddard III, Y. Huang, X. Duan, Monolayer atomic crystal molecular superlattices. *Nature* **555**, 231–236 (2018).

13. N. Wang, H. Tang, M. Shi, H. Zhang, W. Zhuo, D. Liu, F. Meng, L. Ma, J. Ying, L. Zou, Z. Sun, X. Chen, Transition from ferromagnetic semiconductor to ferromagnetic metal with enhanced Curie temperature in $Cr_2Ge_2Te_6$ via organic ion intercalation. *J. Am. Chem. Soc.* **141**, 17166–17173 (2019).

14. Z. Li, X. Zhang, X. Zhao, J. Li, T. S. Herng, H. Xu, F. Lin, P. Lyu, X. Peng, W. Yu, X. Hai, C. Chen, H. Yang, J. Martin, J. Lu, X. Luo, A. H. Castro Neto, S. J. Pennycook, J. Ding, Y. Feng, J. Lu, Imprinting ferromagnetism and superconductivity in single atomic layers of molecular superlattices. *Adv. Mater.* **32**, e1907645 (2020).

15. A. Rousuli, H. Zhang, K. Zhang, H. Zhong, R. Feng, Y. Wu, P. Yu, S. Zhou, Induced anisotropic superconductivity in ionic liquid cation intercalated 1T-$SnSe_2$. *2D Mater* **8**, 015024 (2020).

16. H. Zhang, A. Rousuli, S. Shen, K. Zhang, C. Wang, L. Luo, J. Wang, Y. Wu, Y. Xu, W. Duan, H. Yao, P. Yu, S. Zhou, Enhancement of superconductivity in organic-inorganic hybrid topological materials. *Sci. Bull.* **65**, 188–193 (2020).

17. H. Zhang, A. Rousuli, K. Zhang, L. Luo, C. Guo, X. Cong, Z. Lin, C. Bao, H. Zhang, S. Xu, R. Feng, S. Shen, K. Zhao, W. Yao, Y. Wu, S. Ji, X. Chen, P. Tan, Q.-K. Xue, Y. Xu, W. Duan, P. Yu, S. Zhou, Tailored Ising superconductivity in intercalated bulk $NbSe_2$. *Nat. Phys.* **18**, 1425–1430 (2022).

18. S. Husremović, C. K. Groschner, K. Inzani, I. M. Craig, K. C. Bustillo, P. Ercius, N. P. Kazmierczak, J. Syndikus, M. Van Winkle, S. Aloni, T. Taniguchi, K. Watanabe, S. M.

Griffin, D. K. Bediako, Hard ferromagnetism down to the thinnest limit of iron-intercalated tantalum disulfide. *J. Am. Chem. Soc.* **144**, 12167–12176 (2022).

19. Q. Qian, H. Ren, J. Zhou, Z. Wan, J. Zhou, X. Yan, J. Cai, P. Wang, B. Li, Z. Sofer, B. Li, X. Duan, X. Pan, Y. Huang, X. Duan, Chiral molecular intercalation superlattices. *Nature* **606**, 902–908 (2022).

20. Y. Ahn, G. Lee, N. Noh, C. Lee, D. D. Le, S. Kim, Y. Lee, J. Hyun, C.-Y. Lim, J. Cha, M. Jho, S. Gim, J. D. Denlinger, C.-H. Yang, J. M. Yuk, M. J. Han, Y. Kim, Converting the bulk transition metal dichalcogenides crystal into stacked monolayers via ethylenediamine intercalation. *Nano Lett.* **23**, 9733–9739 (2023).

21. B. Zhou, J. Zhou, L. Wang, J. H. Kang, A. Zhang, J. Zhou, D. Zhang, D. Xu, B. Hu, S. Deng, L. Huang, C. W. Wong, Y. Huang, X. Duan, A chemical-dedoping strategy to tailor electron density in molecular-intercalated bulk monolayer MoS2. *Nat. Synth.* **3**, 67–75 (2024).

22. Z. Bian, Y. Nakano, K. Miyata, I. Oya, M. Nobuoka, Y. Tsutsui, S. Seki, M. Suda, Chiral van Der Waals superlattices for enhanced spin-selective transport and spin-dependent electrocatalytic performance. *Adv. Mater.* **35**, e2306061 (2023).

23. Z. Wan, G. Qiu, H. Ren, Q. Qian, Y. Li, D. Xu, J. Zhou, J. Zhou, B. Zhou, L. Wang, T.-H. Yang, Z. Sofer, Y. Huang, K. L. Wang, X. Duan, Unconventional superconductivity in chiral molecule-TaS_2 hybrid superlattices. *Nature* **632**, 69–74 (2024).

24. Z. Wan, Q. Qian, Y. Huang, X. Duan, Layered hybrid superlattices as designable quantum solids. *Nature* **635**, 49–60 (2024).

25. R. Yang, L. Mei, Z. Lin, Y. Fan, J. Lim, J. Guo, Y. Liu, H. S. Shin, D. Voiry, Q. Lu, J. Li, Z. Zeng, Intercalation in 2D materials and in situ studies. *Nat. Rev. Chem.* **8**, 410–432 (2024).

26. A. R. Ubbelohde, G. S. Parry, D. Nixon, Order–disorder transformations in graphite nitrates. *Nature* **206**, 1352–1354 (1965).

27. X. Xi, L. Zhao, Z. Wang, H. Berger, L. Forró, J. Shan, K. F. Mak, Strongly enhanced charge-density-wave order in monolayer $NbSe_2$. *Nat. Nanotechnol.* **10**, 765–769 (2015).

28. C. R. Dean, L. Wang, P. Maher, C. Forsythe, F. Ghahari, Y. Gao, J. Katoch, M. Ishigami, P. Moon, M. Koshino, T. Taniguchi, K. Watanabe, K. L. Shepard, J. Hone, P. Kim, Hofstadter's butterfly and the fractal quantum Hall effect in moiré superlattices. *Nature* **497**, 598–602 (2013).

29. A. Devarakonda, A. Chen, S. Fang, D. Graf, M. Kriener, A. J. Akey, D. C. Bell, T. Suzuki, J. G. Checkelsky, Evidence of striped electronic phases in a structurally modulated superlattice. *Nature* **631**, 526–530 (2024).

30. C. Wan, X. Gu, F. Dang, T. Itoh, Y. Wang, H. Sasaki, M. Kondo, K. Koga, K. Yabuki, G. J. Snyder, R. Yang, K. Koumoto, Flexible n-type thermoelectric materials by organic intercalation of layered transition metal dichalcogenide TiS_2. *Nat. Mater.* **14**, 622–627 (2015).

31. A. Dasgupta, X. Yang, J. Gao, Naturally occurring van der Waals heterostructure lengenbachite with strong in-plane structural and optical anisotropy. *Npj 2D Mater. Appl.* **5**, 88 (2021).

32. A. Meerschaut, C. Deudon, Crystal structure studies of the 3R-$Nb_{1.09}S_2$ and the 2H-$NbSe_2$ compounds: Correlation between nonstoichiometry and stacking type (= polytypism). *Mater. Res. Bull.* **36**, 1721–1727 (2001).

33. M. B. Walker, R. L. Withers, Stacking of charge-density waves in1Ttransition-metal dichalcogenides. *Phys. Rev. B Condens. Matter* **28**, 2766–2774 (1983).

34. M. S. Dresselhaus, G. Dresselhaus, Intercalation compounds of graphite. *Adv. Phys.* **51**, 1–186 (2002).

35. D. R. Uhlmann, A kinetic treatment of glass formation. *J. Non Cryst. Solids* **7**, 337–348 (1972).

36. C. A. Angell, Formation of glasses from liquids and biopolymers. *Science* **267**, 1924–1935 (1995).

37. P. G. Debenedetti, F. H. Stillinger, Supercooled liquids and the glass transition. *Nature* **410**, 259–267 (2001).

38. A. Gradys, P. Sajkiewicz, E. Zhuravlev, C. Schick, Kinetics of isothermal and non-isothermal crystallization of poly(vinylidene fluoride) by fast scanning calorimetry. *Polymer* **82**, 40–48 (2016).

39. K. Hallavant, M. Soccio, G. Guidotti, N. Lotti, A. Esposito, A. Saiter-Fourcin, Critical cooling rate of fast-crystallizing polyesters: The example of poly(alkylene trans-1,4-cyclohexanedicarboxylate). *Polymers* **16**, 2792 (2024).

40. F. Kagawa, T. Sato, K. Miyagawa, K. Kanoda, Y. Tokura, K. Kobayashi, R. Kumai, Y. Murakami, Charge-cluster glass in an organic conductor. *Nat. Phys.* **9**, 419–422 (2013).

41. S. Sasaki, K. Hashimoto, R. Kobayashi, K. Itoh, S. Iguchi, Y. Nishio, Y. Ikemoto, T. Moriwaki, N. Yoneyama, M. Watanabe, A. Ueda, H. Mori, K. Kobayashi, R. Kumai, Y. Murakami, J. Müller, T. Sasaki, Crystallization and vitrification of electrons in a glass-forming charge liquid. *Science* **357**, 1381–1385 (2017).

42. F. Kagawa, H. Oike, Quenching of charge and spin degrees of freedom in condensed matter. *Adv. Mater.* **29**, 1601979 (2017).

43. H. Oike, A. Kikkawa, N. Kanazawa, Y. Taguchi, M. Kawasaki, Y. Tokura, F. Kagawa, Interplay between topological and thermodynamic stability in a metastable magnetic skyrmion lattice. *Nat. Phys.* **12**, 62–66 (2015).

44. A. de la Torre, Q. Wang, Y. Masoumi, B. Campbell, J. V. Riffle, D. Balasundaram, P. M. Vora, J. P. C. Ruff, G. A. Fiete, S. M. Hollen, K. W. Plumb, Dynamic phase transition in 1T-TaS_2 via a thermal quench. *Nat. Phys.* **21**, 1267–1274 (2025).

45. J. G. Horstmann, E. Hassanpour, A. M. Müller, Y. Zemp, T. Lottermoser, Y. Tokunaga, Y. Taguchi, Y. Tokura, M. C. Weber, M. Fiebig, Dynamic control of ferroic domain patterns by thermal quenching. *Nat. Commun.* **16**, 6802 (2025).

46. M. Yoshida, R. Suzuki, Y. Zhang, M. Nakano, Y. Iwasa, Memristive phase switching in two-dimensional 1T-TaS_2 crystals. *Sci. Adv.* **1**, e1500606 (2015).

47. M. Yoshida, K. Kudo, M. Nohara, Y. Iwasa, Metastable superconductivity in two-dimensional IrTe2 crystals. *Nano Lett.* **18**, 3113–3117 (2018).

48. H. Oike, M. Kamitani, Y. Tokura, F. Kagawa, Kinetic approach to superconductivity hidden behind a competing order. *Sci. Adv.* **4**, eaau3489 (2018).

49. J. V. Riffle, A. de la Torre, K. W. Plumb, S. M. Hollen, Cooling rate dependence of charge density wave phases in 1T-TaS_2 studied by scanning tunneling microscopy and x-ray diffraction. *AIP Adv.* **14**, 105303 (2024).

50. K. Sugimoto, H. Ohsumi, S. Aoyagi, E. Nishibori, C. Moriyoshi, Y. Kuroiwa, H. Sawa, M. Takata, R. Garrett, I. Gentle, K. Nugent, S. Wilkins, Extremely high resolution single crystal diffractometery for orbital resolution using high energy synchrotron radiation at SPring-8. *AIP Conf. Proc.* **1234**, 887–890 (2010).

51. CrysAlisPRO Oxford Diffraction /Agilent Technologies UK Ltd, Yarnton, England. https://agilent.com/cs/library/usermanuals/public/CrysAlis_Pro_User_Manual.pdf.

52. V. Petříček, M. Dušek, L. Palatinus, Crystallographic Computing System JANA2006: General features. *Z. Kristallogr. Cryst. Mater.* **229**, 345–352 (2014).

53. P. Blaha, K. Schwarz, F. Tran, R. Laskowski, G. K. H. Madsen, L. D. Marks, WIEN2k: An APW+lo program for calculating the properties of solids. *J. Chem. Phys.* **152**, 074101 (2020).

54. J. P. Perdew, K. Burke, M. Ernzerhof, Generalized gradient approximation made simple. *Phys. Rev. Lett.* **77**, 3865–3868 (1996).

55. M. Marezio, P. D. Dernier, A. Menth, G. W. Hull Jr., The crystal structure of NbSe2 at 15°K. *J. Solid State Chem.* **4**, 425–429 (1972).

56. D. Zagorac, H. Müller, S. Ruehl, J. Zagorac, S. Rehme, Recent developments in the Inorganic Crystal Structure Database: Theoretical crystal structure data and related features. *J. Appl. Cryst.* **52**, 918–925 (2019).

57. Y. Zhang, D. Zhao, Q. Wang, J. H. Smet, In situ Raman spectroscopy across superconducting transition of liquid-gated MoS_2. *Appl. Phys. Lett.* **120**, 053106 (2022).

58. K. Momma, F. Izumi, VESTA 3 for three-dimensional visualization of crystal, volumetric and morphology data. *J. Appl. Cryst.* **44**, 1272–1276 (2011).

59. J. A. Wilson, F. J. Di Salvo, S. Mahajan, Charge-density waves and superlattices in the metallic layered transition metal dichalcogenides. *Adv. Phys.* **24**, 117–201 (1975).

60. C. Zhang, C.-P. Chuu, X. Ren, M.-Y. Li, L.-J. Li, C. Jin, M.-Y. Chou, C.-K. Shih, Interlayer couplings, Moiré patterns, and 2D electronic superlattices in MoS_2/WSe_2 hetero-bilayers. *Sci. Adv.* **3**, e1601459 (2017).

61. I. Leven, T. Maaravi, I. Azuri, L. Kronik, O. Hod, Interlayer potential for graphene/h-BN heterostructures. *J. Chem. Theory Comput.* **12**, 2896–2905 (2016).

62. S. K. Jain, V. Juričić, G. T. Barkema, Structure of twisted and buckled bilayer graphene. *2D Mater.* **4**, 015018 (2016).

63. Y. Gao, F. Deng, R. He, Z. Zhong, Spontaneous curvature in two-dimensional van der Waals heterostructures. *Nat. Commun.* **16**, 717 (2025).

64. K. Uchida, S. Furuya, J.-I. Iwata, A. Oshiyama, Atomic corrugation and electron localization due to Moiré patterns in twisted bilayer graphenes. *Phys. Rev. B Condens. Matter Mater. Phys.* **90**, 155451 (2014).
